# From GWAS Signals to Causal Genes in Chronic Kidney Disease

**DOI:** 10.3390/cimb48020148

**Published:** 2026-01-28

**Authors:** Charlotte Delrue, Reinhart Speeckaert, Marijn M. Speeckaert

**Affiliations:** 1 Department of Nephrology, Ghent University Hospital, 9000 Ghent, Belgium; charlotte.delrue@ugent.be; 2 Department of Dermatology, Ghent University Hospital, 9000 Ghent, Belgium; reinhart.speeckaert@ugent.be; 3 Research Foundation-Flanders (FWO), 1000 Brussels, Belgium

**Keywords:** chronic kidney disease, genome-wide association studies, causal gene identification, functional genomics

## Abstract

Genome-wide association studies (GWAS) have transformed the study of chronic kidney disease (CKD) by identifying hundreds of genetic loci associated with multiple aspects of kidney function, including albuminuria and CKD risk factors, in diverse populations. A major challenge is translating statistically significant signals into causal genes and mechanisms, as most CKD-associated variants lie in non-coding regulatory regions and often act in a cell type- and context-specific manner. In this review, we provide an overview of the current strategies for moving from GWAS signals toward the identification of causal genes for CKD. We discuss advances in four areas: statistical and functional fine-mapping, molecular quantitative trait locus (QTL) mapping, colocalization, and transcriptome-wide associations, highlighting the advantages and disadvantages of each. We further examined how emerging kidney-specific single-cell, single-nucleus, and spatial transcriptomic atlases have enabled the mapping of genetic risk to specific renal cell types and microanatomical niches. By combining these approaches with chromatin interaction data, multi-omics analytics, and clustered regularly interspaced short palindromic repeats (CRISPR)-based studies, the process of generating causal relationships and mechanistic understanding has been further refined. Importantly, this review provides a unifying framework that synthesizes cross-sectional and longitudinal GWAS with kidney-specific functional genomics to distinguish genetic determinants of CKD susceptibility from modifiers of disease progression, thereby highlighting how regulatory variation and disease trajectories inform precision nephrology. As a result, we can provide insights into the role of genetically informed gene prioritization for experimentation, therapeutic target discovery, and the development of a framework for precision nephrology. Together, these advancements highlight how human genetics, in conjunction with functional genomics and experimental biology, can link an association signal to a clinically relevant interpretation of CKD.

## 1. Introduction

Chronic kidney disease (CKD) affects over 10% of the world’s population, making it one of the most common causes of cardiovascular disease, death from age-related conditions, and increased costs to the world’s healthcare system. CKD encompasses a wide variety of disorders with diverse potential causes and mechanisms, creating distinct molecular pathways by which CKD progresses in individuals. Consequently, the common clinical diagnostic definitions of CKD, the patient’s estimated GFR (eGFR), and albuminuria do not provide the level of understanding and insight into the disease process needed to advance new research that improves our ability to treat CKD with effective therapies [1]. 

Genome-wide association study (GWAS) data collected over the past decade have greatly improved our understanding of the genetic influences on both kidney function and CKD. This has provided insight into the numerous genetic loci associated with eGFR, albuminuria, and the risk of CKD, facilitating further research into new therapeutic methods [1,2]. GWAS findings demonstrate the contribution of common genetic variations to kidney function and disease risk and identify many biological pathways that have not been previously associated with renal pathophysiology. However, despite the notable successes of GWAS, identifying causal genes and mechanisms from GWAS data remains a challenging task. Most variants associated with CKD are located within noncoding regions of the human genome, most of which are within regulatory regions and exert effects on the transcriptional control of distant genes. Consequently, the gene nearest to a GWAS lead variant is frequently not the true effector gene. In addition, linkage disequilibrium can mask the causal variants. Simultaneously, kidney-relevant regulatory genes are often highly cell-type-specific and context-dependent, limiting the interpretability of GWAS signals in bulk tissue or blood-derived datasets [3]. A central challenge in translating these genetic discoveries is that the vast majority of CKD-associated variants lie in non-coding regulatory regions, making it difficult to identify which genes they regulate and in which kidney cell types and disease contexts these regulatory effects occur.

Researchers are developing solutions to address these challenges by using the latest methods and technologies in functional genomics and statistical genetics. Molecular quantitative trait locus (QTL) analysis, colocalization analysis, and transcriptome-wide association studies (TWAS) have enabled us to systematically prioritize candidate genes that are likely to be causal at the GWAS loci. Additionally, comprehensive single-cell and spatial transcriptome maps of the human kidney provide previously unavailable high-resolution data on gene expression across renal cell types and disease-associated states of the kidney. These data also enable researchers to map genetic associations with specific nephron segments and cellular compartments, thereby providing vital information about the biology of CKD [4,5]. In conjunction with multi-omics approaches and precise clustered regularly interspaced short palindromic repeats (CRISPR)-based gene manipulation, this information is changing the interpretation and confirmation of GWAS results. The use of human genetics as a natural experiment is increasingly informing causal inference, therapeutic target selection, and drug development efforts for a wide variety of complex disease conditions, including CKD [6]. In this review, we discuss the current strategies for translating GWAS signals into causal genes in CKD, highlight key methodological advances, and outline the remaining challenges and opportunities for translating genetic discoveries into biological insights and clinical impacts.

## 2. Materials and Methods

This narrative review provides a comprehensive overview of the literature concerning GWAS, post-GWAS strategies, kidney disease-specific functional genomics, and experimental studies for the causal identification of genes involved in CKD. It was not performed using a formalized systematic or scoping review method, nor were there any predefined inclusion or exclusion parameters or quantitative evidence synthesis employed. Literature searches were conducted using PubMed, Web of Science, and Google Scholar to identify relevant publications through October 2023. The search terms used were combinations of words related to CKD, GWAS, fine-mapping of GWAS, molecular QTLs (i.e., eQTLs, sQTLs, and pQTLs), transcriptome-wide association studies (TWAS), single-cell and spatial transcriptomics, chromatin interaction mapping, and functional validation studies. Additionally, the reference lists of key articles, large consortium articles, and high-impact reviews were reviewed. 

Studies in this narrative review were selected based on the following criteria: (1) they provided additional insight into the genetic basis of chronic kidney disease, (2) they presented methods of translating GWAS findings into possible causal genes, (3) they presented functional genomic resources that are specific to the kidney and/or cell types, and (4) they evaluated both the experimental validation of identified causal genes and the translational implications associated with these genes. All selected studies were required to involve large sample sizes from GWAS, utilize kidney-related molecular data, include information regarding the longitudinal duration or focus of the study, and provide a valid example with strong genetic and/or functional support.

Using qualitative methods, the literature reviewed was assessed to identify key biological concepts, common themes, strengths and weaknesses of methods used, and potential future focuses for precision nephrology, to help refine the development of precision nephrology methodology. This review is narrative in nature. Therefore, the studies cited are not intended to represent all possible research but instead reflect what has been deemed important by experts in this area of clinical research.

## 3. GWAS Discoveries in CKD: What Have We Learned?

GWAS has revolutionized our understanding of CKD and its genetic basis by allowing large-scale, adequate searches for common variants related to kidney function. In the early days of GWAS, limited power and sample size constrained the number of variants identified as significantly associated with CKD. However, thanks to the creation of large international collaborative databases and biobanks, numerous studies have brought together thousands of individuals, producing powerful and comprehensive meta-analyses across multiple genetic datasets, with hundreds of thousands to over a million subjects, resulting in many hundreds of unique genetic loci related to CKD. Together, these studies have shown that CKD is a highly polygenic trait, and that each genetic locus has a small individual effect but a cumulatively substantial contribution to differences in individuals’ CKD risk [1]. 

### 3.1. Key CKD-Related Traits and Major Loci

The phenotype most extensively studied is the eGFR calculated from serum creatinine concentration (eGFR_crea_) because it is clinically utilized in clinical practice and is obtainable from large population cohorts. GWAS of eGFR have consistently identified multiple genomic loci in proximity to genes related to renal tubular solute handling, including *UMOD*, *SLC34A1*, and *SLC22A2*, nephron development and structure, including *HNF1B* and *PAX2*, and metabolic pathways and mitochondrial function [1,2]. Several of these genomic loci are co-located with genes associated with monogenic forms of kidney disease, supporting the relevance of GWAS findings and suggesting that both rare and more common forms of kidney disease share some biological mechanisms.

The greatest advancement in eGFR genetics to date is the result of a combined multi-ethnic GWAS meta-analysis with a sample size that exceeds 1 million subjects [2]. This GWAS meta-analysis replicated 264 separate eGFR loci, including 166 new loci. It demonstrated that approximately 7% of the phenotypic variation in eGFR could be explained by the combination of variants from these 264 replicated loci and that approximately 20% of the heritability of eGFR could be accounted for by these GWAS results, thus confirming that kidney function is an extremely polygenic trait. Effect estimates were largely consistent across different ancestry groups, indicating that many common genetic determinants of kidney function are shared globally, while simultaneously facilitating improved locus discovery through increased power. Importantly, the validity of such large-scale trans-ancestry analyses critically depends on the strict control of population stratification, as even subtle ancestry-related allele frequency differences can lead to confounded genotype-phenotype associations in highly polygenic traits, such as eGFR.

Recent large-scale longitudinal GWAS have shown that the genetic determinants of kidney function decline form a biologically distinct phenotype that is more closely related to clinical outcomes. A meta-analysis of 62 longitudinal studies, including more than 340,000 individuals, identified 12 genome-wide significant variants across 11 loci associated with annual eGFR decline, of which nine showed robust, “genuine” associations independent of baseline eGFR [1]. Importantly, all decline-associated loci were previously known from cross-sectional eGFR GWAS, but only a subset of these loci influenced the slope of kidney function loss, highlighting that not all eGFR-associated variants contribute to CKD progression. Loci associated with eGFR decline implicated genes involved in tubular biology and cellular stress responses, with particularly strong evidence for *UMOD*, *GALNTL5*, *SPATA7*, and *TPPP* genes. Several of these genes showed kidney tissue-specific expression quantitative trait locus (eQTL) effects in tubulointerstitial tissue, supporting a regulatory mechanism underlying their association with kidney function decline. Notably, variants at these loci showed larger effect sizes in individuals with diabetes or established CKD, with genetic effects on eGFR decline being two- to four-fold greater in these high-risk groups. This underscores their relevance for disease progression rather than baseline kidney function alone.

To differentiate loci reflecting true kidney filtration from those driven by creatinine metabolism, large cross-sectional studies have systematically integrated GWAS of blood urea nitrogen (BUN), a complementary kidney function biomarker, into their analyses. Of the 264 eGFR-associated loci, 147 showed inverse and statistically significant associations with BUN and were classified as likely kidney-relevant. This integrative biomarker approach demonstrated that the majority of eGFR GWAS signals reflect renal filtration biology rather than extrarenal creatinine generation or secretion [2].

GWAS of albuminuria also shows differences when using the urine albumin-creatinine ratio (UACR). In fact, the loci associated with albuminuria are enriched for all pathways that have been defined as critical for maintaining the integrity of the glomerular filtration barrier, endothelial function, and inflammatory signaling. The loci associated with albuminuria overlap with those associated with eGFR only to a limited degree [1,6]. Consequently, the distinction between lesions of glomerular dysfunction due to glomerular injury and those that lead to loss of GFR supports the fact that these two processes, while related, can be treated as distinct entities. Therefore, a complete understanding of CKD development cannot be achieved solely by relying on eGFR values.

Large GWAS meta-analyses of albuminuria further demonstrated that the genetic effects on UACR are strongly context-dependent, with pronounced gene–diabetes interactions. While variants in the *CUBN* locus represent the only significant genome-wide association with UACR in the general population, their effects were approximately fourfold stronger in individuals with diabetes than in those without diabetes. In diabetes-stratified analyses, two additional loci (*RAB38*/*CTSC* and *HS6ST1*) were identified and independently replicated, with each additional minor allele conferring a 13% decrease (*RAB38*/*CTSC*) or a 21% increase (*HS6ST1*) in mean UACR specifically among individuals with diabetes but not among non-diabetic individuals. Functional follow-up studies have provided strong biological support for these findings. RAB38 expression was significantly increased in the tubular compartments of patients with diabetic kidney disease compared to healthy controls, and both RAB38 and HS6ST1 were preferentially expressed in human tubuli relative to glomeruli. In a streptozotocin-induced diabetic rat model, *Rab38* knockout animals developed markedly increased albuminuria compared to congenic and transgenic controls, despite similar levels of hyperglycemia. This phenotype was accompanied by reduced surface expression of the endocytic receptors megalin and cubilin in proximal tubular cells, indicating that impaired tubular albumin reabsorption is a mechanistic driver of albuminuria. Together, these findings demonstrate that common genetic variations can modulate albuminuria through tubular pathways that become pathogenic in the diabetic milieu [6].

Fine-mapping and post-GWAS analyses of large eGFR datasets have substantially refined causal inferences at many loci. Conditional and Bayesian fine-mapping approaches resolved 58 loci into small credible sets of five or fewer variants, including 20 loci with single-variant resolution. Missense variants with high posterior probabilities were identified in 11 genes, including *SLC47A1*, *SLC22A2*, *CACNA1S*, *CPS1*, and *CERS2*, directly implicating protein-coding changes in kidney function. Several of these genes encode transporters expressed in proximal tubular cells and are involved in drug handling and toxin excretion, highlighting clinically relevant mechanisms linking kidney function, genetics, and pharmacogenomics [2].

Longitudinal genetic analyses showed that variants linked to faster eGFR decline often demonstrate age-dependent effects on cross-sectional eGFR. This is consistent with a concept in which genetic risk increases with aging and cumulative renal stress. Genetic risk scores based on eGFR decline variants are associated with an increased risk of kidney failure and acute kidney injury (AKI), providing direct evidence that progression-associated loci capture clinically meaningful biology rather than measurement artefacts [1].

A simultaneous location (co-localization) analysis of the eGFR GWAS for the 46 different tissues studied (i.e., microdissected human glomerulus, microdissected tubulointerstitium, etc.) yielded 17 genes with evidence of causal overlap. Some of these genes, including *UMOD*, were previously identified to have causal roles and are physiologically significant. Newly prioritized candidates included *FGF5* and *KNG1*, indicating a regulatory influence on kidney-specific cellular contexts. At several loci, regulatory variants are mapped to open chromatin regions in primary tubular or glomerular cells, further supporting a non-coding, cell-type-specific mechanism of action [2].

Recently, GWAS have begun to investigate longitudinal and clinically proximal phenotypes, including the eGFR slope, CKD progression, and kidney failure. Although these analyses remain less powerful than cross-sectional studies, the availability of large longitudinal datasets demonstrates that progression-associated loci preferentially implicate pathways related to fibrosis, inflammation, tubular injury, and maladaptive repair, pointing to biological mechanisms specifically relevant to disease worsening rather than disease onset [1,2].

Analyses across multiple ancestries helped to refine and strengthen CKD-associated loci, particularly those with ancestry-specific effects, and improved causal interpretation by evaluating genetic associations in diverse populations. A key example is the *APOL1* locus, where the G1 and G2 risk variants, which are highly concentrated within individuals of African ancestry, cause a significantly higher risk of CKD progression and kidney failure by causing damage to podocytes. The bulk of the loci associated with kidney function, however, are largely common to all populations but will have improved fine-mapping for these loci when populations have different linkage disequilibrium (LD) structure and patterns of LD. Important examples of this phenomenon include *UMOD*, *HNF1B*, *SHROOM3*, and *SLC22A2*. These loci illustrate how ancestry-informed analyses can refine causal interpretation through differences in LD structure. Overall, these examples show that the genetic architecture for CKD is largely similar for all populations. However, ancestry-informed analyses provide an opportunity for biological insight and the potential for unbiased use of genetic discoveries for all populations [1,7,8,9,10,11,12,13]. Methodological studies of population stratification stress the value of approaches such as principal component analysis, linear mixed models, and ancestry-aware association tests to reduce confounding effects in these complex studies, especially among mixed-ancestry populations and studies that deploy consortium-based meta-analyses. When proper precautions are not taken to manage population stratification, it can generate both false positive associations and miscalculate heritability estimates, indicating the necessity of well-supported statistical methods to efficiently identify CKD risk loci in multicultural contexts.

**Figure 1:**
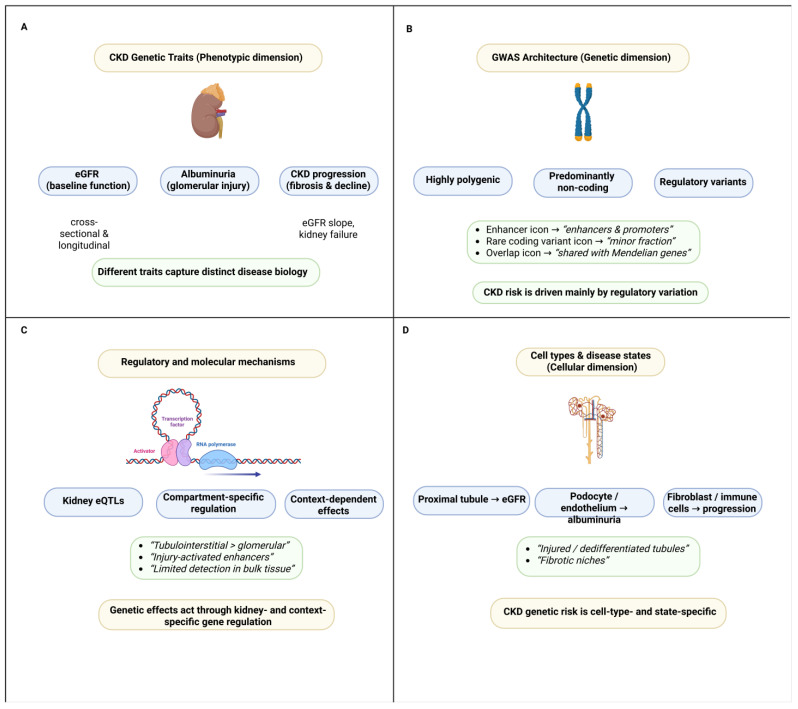
The multidimensional genetic architecture of chronic kidney disease (CKD). (**A**) Overview of CKD-related traits captured in genetic studies, illustrating the phenotypic diversity and interrelated clinical measures used to define disease risk. (**B**) Regulatory characteristics of CKD-associated GWAS loci, highlighting the predominance of noncoding variants and their putative effects on gene regulation. (**C**) Cell type–specific contexts through which genetic risk is mediated, showing enrichment of CKD risk variants in regulatory elements active in relevant kidney cell populations. (**D**) Disease state–specific regulatory landscapes, illustrating how genetic risk effects may differ between healthy and CKD conditions.

Figure 1 summarizes the multidimensional genetic architecture of CKD, including the relationships between CKD-related traits, regulatory aspects of CKD GWAS loci, and the cellular and disease-related contexts in which the genetic risk for CKD appears to be present.

### 3.2. Strengths and Limitations of GWAS in Kidney Disease

A major strength of GWAS is its large scale, high reproducibility, and hypothesis-free design, which allows for the robust identification of disease-associated loci without prior assumptions about biology. Large international consortia and biobank-scale datasets have facilitated meta-analyses involving hundreds of thousands to over one million individuals, yielding highly reproducible associations across independent studies and populations [12,14]. Such large-scale efforts have established kidney function and CKD as highly polygenic traits, with hundreds of loci jointly explaining a substantial proportion of heritability and providing an unbiased entry point into disease-relevant biology of CKD. Aggregation of signals across loci has revealed enrichment in kidney-expressed genes and regulatory elements, providing insight into relevant cell types, particularly tubular epithelial cells. This has created a framework for systematic downstream analyses, including fine-mapping, colocalization, and transcriptome-wide association studies, using kidney-specific expression and epigenomic reference panels [12,15,16,17].

The increase in the availability of kidney-related molecular datasets has greatly increased the utility and value of GWAS in both its ability to describe the association of alleles with disease and its potential for future translation into clinical practice. By linking the genetic associations identified by GWAS with human kidney eQTL atlases, researchers have found that most variants associated with CKD act primarily through non-coding regulatory mechanisms in contrast to causing changes in the function of proteins. Furthermore, the location of the effects of these variants is often specific to the cellular and/or tissue compartment in which they are expressed. Kidney-specific and compartment-resolved molecular analyses consistently indicate that CKD GWAS loci preferentially act through regulatory mechanisms in the tubulointerstitial compartment, particularly proximal tubular cells. At the gene level, these analyses have prioritized several biologically coherent candidates, including *DAB2*, *LRP2*, *CUBN*, *SLC34A1*, SLC22A2, and *UMOD*, all of which are highly expressed in proximal tubular cells and implicated in protein reabsorption, transporter function, or tubular stress responses. Regulatory variants located within the same area (locus) as these regulatory elements may influence the regulation of other genes in the kidneys via a kidney-specific (eQTL) expression quantitative trait locus (i.e., an eQTL with kidney specificity). However, most of these genes cannot be identified using resources such as the Genotype Tissue Expression (GTEx) database that includes tissue from multiple body sites. This highlights the necessity for kidney-derived (or kidney-based) molecular reference panels. 

*DAB2* is a prime example where several studies have shown (1) that eQTLs map to distal enhancers of kidney-specific expression; (2) that tubule-selective loss of DAB2 (via genetic alterations or knockdown) results in a decreased fibrotic response, as well as protection from further renal injury (by preserving renal functioning). Moreover, DAB2 eQTLs demonstrate the utility of genetic evidence from the next generation of genomic methods to directly link GWAS loci for CKD progression with genetic risk [15,16].

However, GWAS alone is insufficient to establish causality, necessitating downstream functional and regulatory analyses [18]. As a result, the gene nearest to a lead single-nucleotide polymorphism (SNP) is frequently not the true effector gene, a limitation repeatedly demonstrated by fine-mapping and functional follow-up studies in the kidney and other complex traits [12,19]. Generalizable fine-mapping frameworks that integrate genetic association data with epigenomic annotations have shown that >90% of likely causal variants map to enhancers active in disease-relevant cell types, highlighting the need for a functional genomic context to interpret GWAS findings. Phenotypic limitations further complicate interpretation. eGFR_crea_ is influenced by muscle mass and creatinine metabolism, albuminuria exhibits substantial intra-individual variability, and the clinical diagnosis of CKD aggregates diverse etiologies with distinct molecular drivers [1,6]. Misclassification and phenotypic heterogeneity reduce statistical power and may obscure subtype-specific genetic effects, particularly glomerular versus tubulointerstitial disease mechanisms [6,20]. 

Longitudinal phenotyping represents another and continuing significant challenge in the GWAS of CKD. Longitudinal measures of kidney function (e.g., decline in eGFR, progression to advanced CKD, and/or progression to kidney failure) are clinically more relevant than cross-sectionally measured eGFR, but the challenges associated with repeated measures of kidney function have historically resulted in GWAS of these phenotypes being underpowered. Logistic complexity, cost, and the heterogeneous nature of the repeated measures of eGFR over time have limited the ability to conduct GWAS of longitudinally phenotyped individuals. Therefore, analyses of longitudinal data require standardized intervals between evaluations, appropriate methods to model non-linear trajectories of kidney function, and adjustment for informative drop-outs, which together have reduced sample size and statistical power compared with cross-sectional studies. The availability of large-scale longitudinal meta-analyses developed over the last years is beginning to provide solutions to the barriers associated with the GWAS of CKD and longitudinal phenotyping. For example, harmonization of cohorts through the use of mixed-effects models (in the case of longitudinally measured eGFR) and slope-based models of eGFR decline allows multiple cohorts to be integrated into large samples. This research has demonstrated that the genetic variants associated with longitudinal decline in eGFR have some but not complete overlap with the genetic variants associated with cross-sectional eGFR at baseline, in that only a minority of cross-sectional eGFR loci also associate with longitudinal decline in eGFR. However, several loci (e.g., *UMOD*, *GALNTL5*, *SPATA7*, and *TPPP*) have been consistently associated with longitudinal decline in eGFR and appear to implicate different pathways involved with tubular biology, cellular stress responses, and maladaptive repair. These findings suggest that genetic risk for progression is increased in the context of metabolic stress or established kidney injury, highlighting gene–environment and gene-disease interactions that are not captured by cross-sectional analyses [1]. The vast majority of GWASs conducted on advanced CKD and kidney failure, although undoubtedly important, have been significantly underpowered. It has been reported by many researchers that, due not only to lower prevalence, but also due to the existence of competing risks and survival bias, the effective sample size will be significantly reduced, reducing the likelihood of identifying variants associated with advanced CKD and kidney failure progression. This results in a significant disadvantage for identifying additional loci that are likely to play a role in the progression of moderate CKD to kidney failure. Overcoming these limitations will require continued expansion of longitudinal cohorts, integration of electronic health record-derived phenotypes, and collaborative efforts to assemble sufficiently powered case–control datasets for kidney failure [1,21].

Additional limitations include the historical overrepresentation of individuals of European ancestry, which limits generalizability and reduces fine-mapping resolution in other populations, as well as residual confounding from population stratification and hidden relationships in very large datasets [7,14]. Despite recent improvements, individuals of African, Latin American, Indigenous, and other underrepresented ethnicities remain markedly underrepresented in GWAS, raising concerns about equity, transferability of polygenic risk scores, and missed opportunities for discovery, driven by diverse linkage disequilibrium patterns. While modern GWAS routinely apply principal component adjustment and linear mixed models, subtle stratification may still bias effect estimates for highly polygenic traits, such as eGFR [7].

The use of genetic information for precision medical treatment and the translation of those genes into precision nephrology research are both impacted by the ethical and equity concerns associated with ancestry bias in GWAS. Most GWAS have focused on individuals who self-identify as Europeans. As such, GWAS results are limited because they do not apply to people of all ancestries, especially in light of the high incidence and severity of CKD in those who identify as African, Hispanic/Latino, Indigenous, or South Asian and the potential for ancestry bias, as shown in the polygenic risk scores and treatment targets. Additionally, the lack of diverse populations in currently available resources that support functional/genomic research (for example, kidney-specific eQTLs and single-cell datasets) significantly limits the ability to make causal inferences about the functional architecture of populations other than those represented in these resources. To solve this problem, we must create a more inclusive approach to GWAS research, carry out GWAS research across multiple ancestries, and create kidney-specific molecular reference panels made up of multiple ancestries so that the findings can be applied fairly to everyone. Despite these limitations, GWAS have provided an indispensable framework for CKD genetics, consistent with broader post-GWAS frameworks describing the transition from association to biological mechanisms and clinical translation across complex diseases [22]. The emerging use of integrative multi-omics has significantly enhanced both the ability to develop knowledge related to CKD risk factors and their ability to identify causative genes, understand the mechanisms associated with CKD, and eventually translate these findings into new therapies for treating kidney disease. GWAS findings combined with information on CKD from kidney transcriptomics, epigenomics, proteomics, metabolomics, and single-cell RNA sequencing have been successful in reclassifying CKD into biologically based subtypes, improving the ability to assess the risk of CKD and to define the therapeutic targets that are specific to the cell types affected by CKD. GWAS findings, when combined with kidney-specific eQTL and chromatin accessibility information, single-cell transcriptomics, longitudinal phenotyping, and experimental validation, will continue to provide insights into biologically significant pathways and therapeutic targets associated with CKD progression [15,16,19,23].

### 3.3. Genetic Modifiers of CKD Progression and Trajectory-Based Disease Classification

CKD is increasingly recognized as a heterogeneous clinical syndrome rather than a single disease entity, encompassing multiple etiologies, molecular mechanisms, and rates of progression. Consistent with this concept, large-scale GWAS have demonstrated that CKD does not have a common genetic “initiator” gene. Instead, CKD risk and progression arise from the cumulative effects of numerous common variants with small individual effect sizes, acting in a highly polygenic and context-dependent manner [1,2,6]. Monogenic kidney diseases, such as autosomal dominant polycystic kidney disease (ADPKD), represent distinct clinical entities with clearly defined causal genes and are, therefore, typically excluded from common variant GWAS of CKD.

Beyond disease susceptibility, longitudinal GWAS have revealed that a subset of genetic loci specifically influences the rate of decline in kidney function rather than the baseline eGFR. These progression-associated loci only partially overlap with cross-sectional eGFR loci and implicate biological pathways related to tubular injury, cellular stress responses, inflammation, and fibrotic remodeling [1]. Importantly, most variants associated with accelerated CKD progression reside in non-coding regulatory regions, particularly enhancers, and exert their effects by modulating gene expression rather than through protein-altering mutations [2,24]. These regulatory variants are frequently active in different renal cell types, especially proximal tubular epithelial cells and interstitial compartments, and often display context-dependent effects that become apparent during injury, metabolic stress or established CKD. The existence of such genetically defined “modifier” or “facilitator” genes provides a plausible biological explanation for the marked interindividual variability in CKD progression observed in clinical practice. Individuals carrying risk alleles that dysregulate the expression of progression-associated genes may experience a more rapid loss of kidney function despite the optimal implementation of established CKD-slowing therapies. An alternative explanation for the observed association of progression-based genetic variants with clinical events is seen in longitudinal genetic studies, which have demonstrated that progression-based genetic variants are significantly associated with clinically adverse events, including renal failure and AKI, in patients carrying these variants, particularly in high-risk patients, such as those with diabetes or pre-existing CKD [1].

It has become evident from these findings that the limitations of existing CKD classification systems, which are based almost exclusively on static thresholds of eGFR and albuminuria, do not adequately reflect the dynamic nature of CKD as the disease progresses. Therefore, an emerging alternative framework is to classify CKD based on longitudinal disease trajectories (i.e., hematuria-first, proteinuria-first, early eGFR decline, or early systemic manifestations, including anemia). Support for this strategy also derives from the genetics and molecular biology of CKD, as different CKD phenotypes (baseline eGFR, albuminuria, and eGFR decline) do not share complete genetic overlap and are regulated through distinct biological pathways and cell types in the kidney [1,6]. For example, albuminuria-associated loci primarily implicate podocyte and glomerular endothelial biology, whereas progression/decline-associated loci have a stronger predominance in tubular and interstitial regulation of kidney disease progression/triggers. 

The combination of trajectory-based phenotyping and kidney-specific functional genomics (such as compartment-resolved eQTLs, single-cell transcriptomics, and spatial transcriptomics) results in biologically informed CKD subtypes. This combination may help improve risk assessment, understand varying responses to treatments, and aid in developing precision nephrology techniques that match the main molecular triggers of a patient’s disease with therapeutic measures. The widespread use of this approach requires large, deeply phenotyped longitudinal cohorts with standardized definitions for trajectories and the ability to integrate clinical, genetic, and molecular data from diverse populations.

## 4. From Associated Variants to Candidate Genes

Identifying the genes and mechanisms that mediate GWAS associations is a central bottleneck in translating human genetics into biological insights for CKD. This challenge is amplified by the regulatory nature of most risk variants, extensive LD, and cellular complexity of the kidney. Recent progress has been driven by the convergence of statistical fine-mapping, functional annotation, molecular QTL analyses, and cell-type-resolved functional genomics, which together provide a principled framework for prioritizing causal genes.

### 4.1. Statistical and Functional Fine-Mapping

GWAS signals typically extend genomic regions containing dozens to hundreds of correlated variants, of which only a small subset is truly causal. Statistical fine-mapping aims to resolve these loci into smaller sets of variants with a high posterior probability of causality. Bayesian approaches, such as approximate Bayes factors and posterior inclusion probabilities, are now standard, enabling the construction of credible sets that quantify uncertainty rather than selecting a single “lead SNP” [25,26,27]. Fine-mapping refines the association signals based on probability instead of position, allowing for the possibility that multiple variants may be collectively associated with the risk of developing a disease while also accounting for the uncertainty caused by LD and sample size limitations. As the sample sizes of CKD GWAS have increased, the ability of fine-mapping to establish more precise locations for causal variants has been established. However, many loci still contain multiple causal variants, which can be attributed to the complexity of LD and sample size limitations.

Fine-mapping performance is strongly influenced by several factors, including the local LD structure, sample size, effect size of causal variants, number of causal variants per locus, and the variant frequency. Even in well-powered studies, the lead SNP is often not the causal variant, particularly when multiple variants in high LD share similar small association statistics. Simulation studies have demonstrated that when effect sizes are small, or LD is strong, posterior probability is distributed across many correlated variants, resulting in large credible sets despite large sample sizes [25]. Importantly, the LD structure itself is population-specific, such that fine-mapping resolution can differ across ancestry groups, even when effect sizes are identical [7].

A major advance has been the use of multi-ethnicity GWAS to improve fine-mapping. Differences in LD structure across populations can break the correlations between variants, substantially narrowing the credible sets and increasing the confidence in causal inference. This approach has proven effective for complex diseases and is particularly relevant for CKD, where Eurocentric LD patterns can conceal causal variants and reduce generalizability [7]. Trans-ethnic fine-mapping is most powerful when ancestries with markedly different LD patterns, particularly African ancestry populations, are included, as shorter LD blocks can substantially reduce the number of candidate causal variants in these populations. However, these gains depend on balanced sample sizes and careful modeling of ancestry-specific effect heterogeneity. Multi-ancestry fine-mapping also addresses equity concerns while simultaneously enhancing biological insights [25]. Failure to properly model global and local ancestry can lead to false associations and incorrect fine-mapping results, as ancestry-correlated variants may be incorrectly prioritized as causal variants [7].

Methodologically, fine-mapping approaches can be broadly categorized into heuristic filtering, penalized regression, and Bayesian variable selection models. While early heuristic approaches relied on LD thresholds or conditional analyses, these strategies were limited by arbitrary cut-offs and reduced power to detect secondary signals. Penalized regression models (e.g., lasso or elastic net) collectively model variants but may preferentially select non-causal determinants in regions with high LD. In contrast, Bayesian fine-mapping methods are specifically designed to estimate the posterior probabilities for each variant and have consistently demonstrated superior performance in identifying causal variants under realistic genetic architectures [25].

When conducting fine-mapping for functional variants, statistical evidence will be adjusted with information from the annotation. Therefore, a way to select regulatory variants is through functional annotations that have been incorporated into a model. Several types of genomic functional annotations include histone modifications, chromatin accessibility, and transcription factor-binding sites. The variant location in predicted enhancer or promoter regions may be increased by using the prior probability of functional annotations under Bayesian methodology. Using a Bayesian approach, functional annotation data may be explicitly included within the prior probability computation of causation for a given variant in the specific cell line associated with the disease of interest. Such approaches modestly but consistently reduce credible set sizes and improve prioritization, particularly in regions of high LD or moderate association strengths [25]. 

Through large-scale bioinformatic fine-mapping studies of loci that regulate kidney function, it has been shown that variants associated with eGFR are particularly abundant in kidney regulatory sites. A detailed analysis of 53 eGFR candidate loci revealed that variants associated with eGFR showed significant overlap with sites of DNase I hypersensitivity and markers of active chromatin (H3K4me3 and enhancers) in adult renal tubular and cortical epithelial cells. No significant overlap was found in non-renal tissues or glomerular endothelial cell types. Chromatin state characterization also confirmed that GWAS variants preferentially localized to weak and strong enhancers in kidney tissues, indicating that changes in regulation in tubular epithelial cells could account for the majority of the genetic effects on kidney function. Functional prioritization based on integrative analyses (DEPICT, GRAIL, and kidney-specific eQT mapping) identified genes involved in tubular transport, endo-lysosomal trafficking, metabolism, and fibrotic signaling. The convergence of evidence from regulatory annotations, enrichment of gene expression in the kidney, and pathway analyses supported several loci, including *DAB2*, *SLC34A1*, *SLC22A2*, *UMOD*, and *LRP2* [1,13].

Importantly, longitudinal GWAS of annual eGFR decline have demonstrated that only a subset of loci identified by cross-sectional eGFR GWAS show real associations with kidney function loss over time, underscoring the value of progression phenotypes for fine-mapping disease-relevant biology. In a meta-analysis of 62 longitudinal studies including over 340,000 individuals, all genome-wide significant variants for eGFR decline mapped to loci previously associated with cross-sectional eGFR. However, only nine variants across eight loci showed “genuine” associations independent of baseline eGFR, distinguishing variants influencing disease progression from those affecting stable baseline filtration levels. This natural filtering of loci substantially refines the set of candidate regions for downstream fine-mapping and functional interrogation. Fine-mapping interpretation in longitudinal GWAS is further complicated by covariate adjustment, particularly for the baseline eGFR. The impact of baseline adjustment on effect sizes and the total number of genome-wide significant loci, while beneficial, resulted in collider bias for some variants classified as significant. To assist in distinguishing true effects (i.e., slope) from incorrect associations, careful phenotype modelling was required in conjunction with statistical fine-mapping to identify the progressive relevant loci and related variants that are still significant when compared to baseline unadjusted models. These considerations are critical when translating fine-mapped variants into mechanistic hypotheses. The integration of longitudinal GWAS fine-mapping with kidney-specific eQTL data enabled the prioritization of candidate causal genes at progression-associated loci. Four genes (*UMOD*, *GALNTL5*, *SPATA7*, and *TPPP*) showed strong in silico functional support, including variant-associated gene expression changes in tubulointerstitial tissue and consistency across baseline-adjusted and unadjusted models. Notably, *GALNTL5*, *SPATA7*, and *TPPP* were also associated with CKD progression among individuals with established CKD at baseline, highlighting how longitudinal fine-mapping can prioritize genes directly relevant to disease worsening rather than disease onset [1].

The integration of fine-mapped GWAS signals with eQTL data provides an additional layer of causal deduction, as GWAS variants are significantly more likely than frequency-matched background variants to act as eQTLs. Statistical colocalization and mediation analyses enable the prioritization of variants that collectively influence gene expression and disease risk, although accurate causal derivation critically depends on the tissue and cell-type relevance of the expression data [25]. eQTL colocalization analyses are particularly sensitive to population stratification and ancestry mismatch between GWAS and molecular QTL datasets, which can bias colocalization probabilities and lead to incorrect causal gene assignment [7]. However, functional fine-mapping remains restricted by the limited availability of kidney- and cell-type-specific epigenomic datasets, especially under disease conditions, highlighting an important gap in the field. The main post-GWAS strategies currently used to move from CKD-associated variants to candidate causal genes, together with their data requirements, strengths, and limitations, are summarized in Table 1.

### 4.2. eQTLs, Colocalization, and TWAS

Although fine-mapping prioritizes variants, identifying the target genes through which these variants act requires additional evidence. eQTL analyses provide a direct link between genetic variation and gene expression and have become a cornerstone of GWAS. Early studies have demonstrated that a substantial proportion of CKD GWAS loci overlaps with eQTLs in human kidney tissue, supporting a regulatory basis for many associations. A major advance came from the generation of one of the first human kidney cortex–specific eQTL maps derived from RNA-seq data in 96 healthy human kidney samples, which identified 1886 genes whose expression was significantly associated with nearby genetic variants (cis-eGenes). This study demonstrated that kidney eQTLs are strongly enriched near transcription start sites and localize preferentially to active promoter and enhancer regions marked by H3K4me1, H3K4me3, and H3K27ac in proximal tubular epithelial cells. This provides direct tissue-relevant evidence that CKD-associated variants act predominantly through gene regulatory mechanisms rather than coding changes [16]. 

The connection between kidney-specific eQTL datasets is substantially better than that of other multi-tissue databases, because of the large proportion of regulatory influences on gene expression in non-renal tissues that are absent or diluted. Comparative research indicates that the majority of CKD-associated genetic variants show co-localization with eQTLs in the kidney, highlighting the importance of using molecular reference panels appropriate for the particular gene and disease being studied [3].

The most recent studies now include splicing QTLs (sQTLs), protein QTLs (pQTLs), and chromatin accessibility QTLs (cQTLs), which reflect the wide variety of ways by which genetic variation produces phenotypic variation. Kidney-specific eQTLs show an increased frequency of association with CKD and eGFR GWAS signals than to non-kidney GWAS variant categories, further illustrating the specificity of kidney-derived gene molecular data type [16]. 

Beyond steady-state gene expression, alternative splicing is increasingly recognized as a mechanism linking genetic variation to kidney disease. Splicing QTLs capture regulatory effects missed by standard eQTL analyses, and several CKD-associated loci influence exon usage rather than total gene expression, suggesting that post-transcriptional regulation is an underappreciated contributor to CKD pathogenesis [3].

Because GWAS and eQTL signals may overlap incorrectly due to LD, colocalization analyses are routinely used to formally test whether the same causal variant underlies both signals. Bayesian colocalization frameworks estimate the posterior probability of a shared causal variant and help distinguish true regulatory mechanisms from coincidental overlaps [3,4]. However, most early colocalization methods assume a single causal variant per locus, an assumption frequently violated in both GWAS and eQTL studies because of widespread allelic heterogeneity [4].

eCAVIAR is a probabilistic colocalization framework that explicitly models multiple causal variants per locus and integrates GWAS and eQTL summary statistics while accounting for LD structure. eCAVIAR computes a colocalization posterior probability (CLPP), defined as the probability that the same variant is causal in both studies, thereby unifying fine-mapping and colocalization within a single statistical framework. Simulation studies have demonstrated that ignoring allelic heterogeneity leads to a systematic underestimation of colocalization probability and inflated false negatives, particularly in regions of complex LD. In contrast, eCAVIAR maintained high specificity and low false-positive rates, even in loci having two or more causal variants, which is common in regulatory regions. Importantly, the magnitude of the CLPP depends strongly on local LD complexity, which explains why true colocalization can result in only modest posterior probabilities in regions of high correlation despite shared causal variants. [4].

The application of Bayesian colocalization to kidney eQTL and CKD GWAS data identified multiple loci with strong evidence of shared causal variants (posterior probability for colocalization > 0.8), including *PGAP3*, *SPATA5L1*, *EEF1AKMT2*, *KNG1*, and *MANBA*. These analyses demonstrated that the gene nearest to the GWAS lead SNP was frequently not the gene whose expression was affected, directly illustrating the limitations of proximity-based gene assignment [16]. The eCAVIAR framework further demonstrated that many GWAS loci show statistically significant signals in both GWAS and eQTL datasets, yet fail to colocalize. This indicates that distinct variants within the same locus independently influence disease risk and gene expression. Such cases conflict with simplistic regulatory interpretations of GWAS loci and underscore the need for formal colocalization testing [4].

Among these loci, MANBA emerged as an interesting example of how eQTL-GWAS colocalization can be used to identify biologically meaningful target genes. Risk alleles associated with reduced kidney function were associated with significantly lower MANBA expression in human kidney tissue, and MANBA expression was also reduced in the kidneys of individuals with CKD and fibrosis. Functional validation in zebrafish demonstrated that knockdown of MANBA resulted in pericardial edema and defective pronephric development. These phenotypes are consistent with impaired renal function, whereas knockdown of the GWAS-suggested nearby gene *NFKB1* did not produce any kidney phenotypes. This provides rare experimental confirmation that eQTL-prioritized genes can represent the true causal mediators of GWAS signals in CKD [16].

TWAS extend these approaches by integrating GWAS summary statistics with genetically predicted gene expression to test for associations between predicted expression levels and disease characteristics. TWAS has been applied to kidney function features and CKD for the identification of candidate genes that may be distal to GWAS lead variants and thus missed by positional mapping [5]. TWAS is fundamentally a gene-level association test rather than a causal deduction framework, and TWAS-significant genes should not be automatically interpreted as causal mediators of GWAS signals. A central limitation is co-regulation: TWAS frequently identifies multiple associated genes per locus because genetically predicted expression levels are correlated across nearby genes. This correlation can arise from shared eQTLs, distinct eQTLs in LD, or overlapping sets of GWAS variants used in the expression prediction models. Consequently, non-causal genes may appear as strong TWAS hits simply because their predicted expression is correlated with that of the true causal gene. Simulation and empirical analyses have demonstrated that TWAS signals often reflect correlated expression rather than direct causal effects [5]. This reinforces the need to interpret TWAS results only in conjunction with fine-mapping and colocalization evidence [4]. Importantly, TWAS associations may be detected even with low predicted expression correlation when expression models are driven by the same GWAS-associated variant or a closely linked variant in strong LD. This mechanism is particularly problematic because it is not readily detectable using simple correlation metrics [5].

However, TWAS does not formally test colocalization and may detect associations driven by LD between several causal variants. Consequently, TWAS signals can arise even when GWAS and eQTL causal variants are different, emphasizing that TWAS associations alone cannot establish regulatory causality [4].

There are several critical limitations of TWAS in kidney disease, including the co-regulation of nearby genes, shared eQTL architecture across loci, and difficulty in assigning directionality between gene expression and disease. There can be a strong tissue bias in TWAS; expression reference panels from tissues that are not mechanistically relevant to the disease can both miss true causal genes and prioritize incorrect associations. Empirical analyses have demonstrated that many literature-supported causal genes lose TWAS significance when expression models are trained in non-relevant tissues, whereas non-causal genes may remain significant because of the shared regulatory architecture [5]. Simulation and empirical analyses have demonstrated that TWAS signals often reflect correlated expression rather than direct causal effects, reinforcing the need to interpret TWAS results only in conjunction with fine-mapping and colocalization evidence [3]. The kidney eQTL study highlights this limitation by showing that many GWAS loci do not colocalize with kidney eQTLs despite strong association signals. These findings indicate that TWAS signals lacking kidney-specific colocalization should be interpreted cautiously and prioritized only when supported by tissue-relevant regulatory evidence [16].

A persistent limitation of eQTL- and TWAS-based approaches is their reliance on bulk tissue reference datasets, which average signals across heterogeneous cell populations. This averaging can mask regulatory effects that are restricted to rare or specialized kidney cell types, motivating a transition toward cell-resolved analyses. Indeed, the kidney cortex eQTLs generated in the attached study largely reflect proximal tubular cells, which dominate the cortical tissue composition. This emphasizes both the strength of detecting tubule-relevant regulation and the need for future single-cell and compartment-specific eQTL maps to capture glomerular, endothelial, immune, and interstitial cell–specific genetic effects in CKD [16]. Emerging strategies, such as TWAS fine-mapping (e.g., FOCUS), multi-tissue modeling, and integration with single-cell expression atlases, are expected to substantially improve causal gene prioritization by explicitly modeling co-regulation and tissue specificity [5].

### 4.3. Kidney Cell-Type Specificity and Regulatory Context

The kidney is one of the most cellularly diverse organs, comprising highly specialized epithelial, endothelial, immune, and stromal cell populations organized into distinct anatomical compartments. Increasing evidence indicates that the genetic risk for CKD is mediated through highly cell-type- and compartment-specific regulatory mechanisms, rather than uniform effects across organs. Early enrichment analyses of eGFR GWAS loci demonstrated preferential expression of prioritized genes in tubular epithelial cells, particularly the proximal tubule. These findings imply that solute transport, metabolic, and endo-lysosomal pathways are central to the genetics of kidney function. Chromatin state and DNase I hypersensitivity mapping further showed that eGFR-associated variants are favorably located within kidney-specific regulatory regions rather than extrarenal tissues, supporting a model of direct intrarenal genetic effects. However, these early analyses relied largely on bulk tissue data and indirect inference, which limited resolution of the specific cellular contexts in which genetic risk operates [13]. 

Single-cell and single-nucleus RNA sequencing has transformed this landscape by allowing the direct mapping of genetic risk to disease-relevant kidney cell types and injury-associated states, rather than bulk tissue averages. Comprehensive maps of the human kidney have identified gene expression across dozens of epithelial, endothelial, immune, and stromal cell types and states using single-cell and single-nucleus RNA sequencing [28,29,30]. These studies demonstrated that kidney function–associated genes are strongly enriched in proximal tubular cells, whereas albuminuria-associated loci preferentially are present in podocytes and glomerular endothelial cells, and progression-related phenotypes implicate fibroblasts and immune populations [31]. Thus, single-cell data independently confirmed that CKD genetic risk is not organ-wide but instead converges on discrete renal cell populations [13,31].

A critical advance beyond descriptive single-cell expression plans was the generation of compartment-resolved human kidney eQTL maps, in which glomerular and tubulointerstitial tissues were microdissected before RNA sequencing. This approach revealed thousands of cis-eQTLs in each compartment with substantial compartment specificity. Many regulatory variants influenced gene expression only in the glomerular or the tubulointerstitial fraction, even when baseline expression of the target gene was similar in both compartments. Importantly, CKD and eGFR GWAS loci were significantly enriched for eQTLs in the tubulointerstitial compartment rather than in the glomeruli, providing direct genetic evidence that much of the common CKD risk is mediated through tubular and interstitial regulatory mechanisms rather than through primary glomerular dysfunction. Tubule-specific eGenes showed particularly strong higher levels of kidney function GWAS variants, whereas compartment-shared eGenes were more frequently associated with non-renal diseases. These findings highlight the disease specificity of compartment-resolved regulation [15].

Integration of compartment-specific eQTLs with epigenomic maps further demonstrated that CKD-associated variants favorably localize to distal enhancer elements active in proximal tubular epithelial cells rather than promoters or coding regions. These enhancers frequently overlap with histone marks associated with active or primed regulatory states (e.g., H3K27ac and H3K4me1) and are largely absent from multi-tissue reference resources. This explains why many kidney-relevant regulatory effects are missed in datasets such as GTEx [15]. Collectively, these findings support a model in which common CKD risk variants act predominantly through cell-type–specific regulatory programs in tubular cells [1,15].

Recently, spatial transcriptomics has added anatomical resolution by preserving tissue architecture while measuring gene expression. Spatial approaches have revealed that genetically prioritized genes often show highly localized expression patterns within specific nephron segments or fibrotic interstitial niches. These patterns are not readily apparent in dissociated single-cell data [30]. These spatially restricted expression domains frequently match regions of tubular injury, inflammation, and fibrosis, linking genetic risk loci to microanatomical contexts central to CKD progression and complementing compartment-resolved eQTL findings [15,30].

The compartment-specific regulatory framework has also allowed the direct functional prioritization of candidate causal genes at GWAS loci. Integration of CKD GWAS with tubulointerstitial eQTLs markedly increased the number of loci for which a plausible causal gene could be identified compared with whole-kidney analyses. Among these, DAB2 emerged as a prototypical tubule-specific causal gene, with risk alleles regulating DAB2 expression exclusively in the tubulointerstitial compartment. The associated variant overlaps a kidney-specific distal enhancer active in proximal tubular cells but inactive in non-renal tissues. DAB2 illustrates how genetically and experimentally validated tubular pathways can serve as high-confidence therapeutic targets [15].

Single-cell and single-nucleus transcriptomic analyses have further refined the interpretation of these regulatory mechanisms by distinguishing mature adult kidney cell states from the developmental programs [25]. Comparative analyses of adult human kidney tissue and pluripotent stem cell-derived kidney organoids have demonstrated that, although organoids recapitulate many renal lineages, their transcriptional profiles remain developmentally immature, with incomplete activation of adult functional and regulatory pathways [31]. Notably, many CKD GWAS-prioritized genes show strong, highly specific expression in mature proximal tubular cells but are weakly expressed or absent in organoid-derived counterparts. This highlights the limitations of developmental models for interpreting adult CKD genetic risk [15,31].

Importantly, the regulatory effects may be context-dependent, manifesting only during injury, inflammation, or fibrotic remodeling. Tubulointerstitial eQTLs preferentially colocalize with regulatory elements active in injured or dedifferentiated tubular states, suggesting that genetic effects may become apparent primarily under disease-relevant conditions [15]. Together, compartment-resolved eQTL mapping, single-cell and spatial transcriptomics, epigenomic annotation, and in vivo functional validation converge on a model in which CKD genetic risk is mediated through highly specific regulatory programs acting in defined tubular and interstitial cell populations and pathological contexts [1,15].

## 5. Emerging Tools to Refine Causal Inference

While GWAS have robustly mapped hundreds of loci associated with kidney function and kidney disease, defining the causality between genetic variation, genes, cell types, and disease mechanisms will require a greater level of resolution. Recently, next-generation technologies have generated substantial advances in this area by addressing three questions: In what cell types is the genetic risk present? What regulatory mechanisms is the genetic risk guided by? Through what molecular pathways is the genetic risk generated? Genomics at the single-cell and spatial resolution, 3D architecture of the genome, and integrated multi-omics will provide the basis for new interpretations and validations of GWAS data related to kidney diseases.

Importantly, each of the emerging tools described in this section directly addresses the key limitations of GWAS and post-GWAS analyses discussed earlier in this review. Single-cell and single-nucleus transcriptomic approaches overcome the averaging effects of bulk tissue analyses by resolving cell-type-specific expression patterns and injury-associated states in which genetic risk manifests. Spatial transcriptomics enables the identification of spatially distinct microanatomical niches affected by CKD-related pathologies (e.g., fibrosis, inflammation, and tubular injury) through genetically prioritized genes using a combination of mapped spatial transcriptomic data and extensive patient-derived genetic data. By combining long-range enhancer–promoter interactions with 3D genome mapping technologies, chromatin changes, and the mapping of chromatin at the genomic level, we can gain a deeper understanding of the genetic contribution to CKD. The difficulties in determining the targets of CKD-related, primarily non-coding variants are addressed by chromatin mapping technologies. The implementation of a multi-omic approach provides a comprehensive understanding of the genetics of CKD, including comprehensive mapping of an individual’s genomic variation and phenotypical response to genetic variation. CRISPR-based genome and epigenome editing enables the functional validation of candidate genes and regulatory elements derived from human studies. Although the use of CRISPR-Cas9 gene editing technology is not limited to research, its use to investigate the functions of candidate genes can establish a direct link between genes and causality. Overall, these technologies provide a comprehensive set of tools to systematically resolve the major methodological and biological barriers associated with translating GWAS results into the causal mechanisms of CKD.

Building on the cell-type-resolved insights described above, newer technologies now enable causal deduction to be refined through spatial, epigenomic, and experimental integration. In addition, researchers have identified many injury-induced and disease-associated cell types. By creating databases with large datasets, researchers can identify genes associated with an increased risk of developing CKD and map these genes to specific nephron segments and areas of the kidney enclosed by the nephron. This mapping demonstrates how CKD-related genes limit the risk of CKD to particular cell types; for example, each gene associated with CKD risk is located in a unique type of nephron cell. Loci associated with eGFR show higher levels of genes in proximal tubular cells, whereas loci associated with albuminuria show enrichment in podocytes and glomerular endothelial cells. Loci related to CKD progression can be linked to higher levels of expression of genes located within fibroblasts and immune cell populations [30,31]. 

Building on these advances, spatial transcriptomics has added a critical anatomical dimension by keeping the spatial organization of gene expression within the kidney tissue. Spatial profiling has proven to be powerful in CKD, where fibrosis, inflammation, and tubular atrophy are heterogeneously distributed in the kidney. Recent spatial maps of human kidney tissue have shown that many genes prioritized by GWAS, eQTL, and single-cell analyses show highly localized expression within fibrotic interstitial regions, injured proximal tubules, and immune-enriched microenvironments [30]. These spatially restricted expression patterns align genetic risk loci with precise microanatomical sites of pathology, strengthening causal deduction by linking regulatory variation to disease-relevant tissue niches that are obscured in dissociated cell datasets.

Another major advance comes from chromatin interaction and 3D genome mapping technologies, which take care of the predominantly non-coding nature of CKD-associated variants. Enhancers can regulate target genes over long genomic distances, making linear proximity an unreliable guide for causality. Chromosome conformation capture-based approaches, including Hi-C and promoter capture Hi-C, enable the direct mapping of physical contacts between distal regulatory elements and gene promoters. The integration of GWAS fine-mapped variants with chromatin interaction data has demonstrated that many kidney-associated variants interact with promoters of genes that are not the nearest in linear distance [3,32]. Although kidney-specific 3D genome maps remain limited in scale, existing studies indicate that even moderate-resolution, tissue-relevant chromatin interaction data substantially improve GWAS-to-gene mapping when combined with epigenomic and eQTL evidence.

Importantly, evidence that converges across several molecular layers strengthens the causal inference relationship, which is why integrative multi-omics techniques have been developed. Multi-omics studies combining genetic data with transcriptomic, epigenomic, proteomic, and metabolomic measurements have begun to reveal how GWAS signals align with coordinated molecular changes across the regulatory and functional layers [15,23]. In CKD, integrative analyses have shown that loci associated with kidney function frequently map to networks regulating tubular metabolism, endo-lysosomal trafficking, inflammatory signaling, and fibrotic remodeling. Computational frameworks, such as joint single-cell RNA–ATAC profiling and multi-omics factor analysis, further enable the separation of shared disease programs (e.g., fibrosis or immune activation) from cell-type–specific regulatory mechanisms.

Finally, experimental modification technologies are increasingly used to functionally validate genetically prioritized regulatory elements and genes. CRISPR-based genome editing and epigenome editing approaches (CRISPR-Cas9, CRISPRi, and CRISPRa) now allow the direct manipulation of non-coding enhancers and candidate effector genes in human kidney cell lines, primary tubular epithelial cells, and pluripotent stem cell–derived kidney organoids [32,33,34,35]. These systems allow the causal testing of GWAS-derived hypotheses by linking specific regulatory modifications to transcriptional, cellular, and phenotypic outcomes. Importantly, the integration of CRISPR modified data with human genetic evidence has already demonstrated that genes supported by convergent GWAS, eQTL, and functional data are more likely to exert disease-relevant effects when mutated [15].

The combination of these new technologies allows GWAS loci to become dynamic mechanisms instead of the simple statistical association they have traditionally been viewed as, by providing a mechanistically definable way of what type of kidney cell they act in, their associated regulatory regions, and the related pathophysiological conditions in which they operate. 

## 6. Experimental Validation and Translational Relevance

While statistical prioritization of candidate causal genes is a crucial starting point for understanding the mechanism of CKD, it is not adequate unless combined with experimental evidence that validates the associated causal relationship(s), determines the directionality of the effects, and shows how genetic modifications lead to cellular dysfunction, tissue injury, or the eventual development of clinical phenotype(s). Additionally, human genetics provides a robust framework for conducting translational research, as it enables investigators to identify biologically relevant pathways and therapeutic targets with a high probability of translating into clinical efficacy. The combination of experimental validation and interpretation within the context of human genetics creates an important platform connecting GWAS discoveries in nephrology to realizable patient benefits.

### 6.1. Functional Validation in Cellular and Animal Models

The growing recognition that most CKD GWAS variants act through regulatory mechanisms has shifted experimental validation away from simple gene knockout strategies toward context-aware mutation models. In vitro systems, particularly human-derived cellular models, have become central to testing the functional consequences of candidate genes and their regulatory elements in vivo.

Human kidney cell lines and primary cells, including proximal tubule epithelial cells, podocytes, and endothelial cells, are frequently used for initial validation because of their scalability and tractability. These models allow the targeted mutation of candidate genes using CRISPR-based approaches (CRISPR-Cas9, CRISPRi/a) to assess their effects on transcriptional programs, cellular stress responses, transport functions, and fibrotic or inflammatory signaling. Importantly, such experiments can test hypotheses generated by eQTLs or enhancer–promoter interactions, for example, by disrupting a non-coding regulatory element rather than the gene body itself. Recent CRISPR-based enhancer mutation studies have demonstrated that non-coding elements prioritized by GWAS and chromatin interaction data frequently exert quantitatively modest but biologically meaningful effects on gene expression [32,33].

Organoids derived from human iPSCs represent a significant development in terms of the ability to validate gene function. In addition, these organoids may serve as excellent models for the validation of genes involved in nephrogenesis, tubular differentiation, and the response to injury in the epithelial compartment. CRISPR techniques have been used in recent studies on organoids to create models of monogenic kidney disorders. The use of genetic modifications allows the modelling of candidate genes identified through GWAS to further establish the relationship between genetic variations and the development of kidney diseases [34,36]. In addition, the development of kidney organoids will provide researchers with a scalable platform for the functional validation of human kidney disease-related genes [36]. The development of high-throughput screening methods has enabled researchers to systematically investigate the mechanisms of genetic modifications across multiple renal lineages. Using this new technology, researchers can now examine the effects of genetic modifications on the proximal tubules, distal segments of the nephron, and podocytes simultaneously [34]. Although the organoids created do not display full vascularization or possess the ability to replicate immune cells, the data collected from the analysis of organoids using single-cell sequencing technology support the use of organoids as a potential model for early kidney tubule injury and repair [31].

Animal studies are necessary to examine the long-term effects of genetic mutations and disruptions on the functions of the entire body. Mouse studies allow researchers to study the development and functioning of the kidneys and the body’s response to injury in an intact animal. To clarify the functions of genes that were determined to be priority targets through GWAS, animal studies have created “knockout” (removal of the gene), “knockin” (addition of a gene), and conditional alleles (gene can be removed at a specific time) of GWAS-targeted genes. Using targeted deletions and additions, researchers have determined how gene function varies by cell type, specifically in tubular epithelial cells and in podocytes. Importantly, several loci implicated by CKD GWAS overlap with genes known to cause Mendelian kidney diseases, providing strong genetic validation and rationale for deeper functional investigation [1]. Beyond developmental phenotypes, inducible and injury-triggered mouse models have proven to be essential for testing whether GWAS-prioritized genes modify susceptibility to fibrosis, inflammation, or maladaptive repair following ischemic or toxic insults, thereby directly linking genetic variation to CKD-relevant pathological processes [13,15].

Additionally, there are major differences between species that affect not only the structural organization of the kidney but also the way in which the genes are regulated and the manner in which diseases occur, especially when considering regulatory variants that use different types of enhancers that have evolved specifically for humans. Currently, there is much more focus on using cross-species triangulation as a way to support the evidence for a causal relationship by considering the consistent effects observed among human cells, organoids, and animal models in an attempt to strengthen the conclusion of causality. This layered strategy, which combines human genetic evidence, CRISPR perturbation in human-derived systems, and mechanistic validation in vivo, has emerged as the best practice framework for establishing causality at CKD GWAS loci and minimizing false-positive functional interpretations [32,35].

### 6.2. Implications for CKD Biology and Drug Discovery

Genetic discoveries from GWAS have fundamentally reshaped our current understanding of CKD biology by revealing disease mechanisms that extend beyond traditional glomerular-centric models. Rather than primarily implicating podocyte injury, GWAS consistently highlights pathways related to tubular transport, metabolic stress responses, endo-lysosomal trafficking, and maladaptive repair [1,13,15]. This genetic evidence supports a paradigm shift in CKD pathophysiology toward tubular-centered disease mechanisms that precede nephron loss and fibrosis.

An important translational implication of GWAS is the discovery of therapeutic targets. Large-scale analyses have demonstrated that drug targets supported by human genetic evidence have a substantially higher probability of regulatory approval than targets lacking such support. Revised estimates indicate that genetic support is associated with a substantially higher likelihood of drug approval, with revised estimates indicating an approximately two-fold enrichment compared with targets lacking genetic evidence [35]. In CKD, where late-stage trial failure is common and disease-modifying therapies remain limited, this provides a strong justification for prioritizing genetically supported targets.

CKD GWAS has already highlighted biologically plausible and potentially druggable pathways. Variants at loci such as *UMOD*, *SLC34A1*, *SLC22A2*, *LRP2*, and *DAB2* are implicated in tubular salt handling, solute transport, endocytosis, and cellular stress signaling [1,13,15]. Several of these genes encode membrane proteins or regulators of intracellular trafficking, pathways that are available for pharmacological modulation, and act upstream of irreversible fibrosis. Importantly, functional studies have demonstrated that modification of these pathways alters susceptibility to tubular injury and progression [13,15].

Human genetics also provides a framework for evaluating the safety and pleiotropy of targets. Comprehensive analyses of genetic variation across diseases have demonstrated that many loci exert context-dependent effects across organ systems, emphasizing the importance of considering pleiotropy during target selection [37]. In CKD, the integration of GWAS findings with phenome-wide association analyses has shown that some kidney-related genes also influence cardiovascular and metabolic traits, which is critical for early risk–benefit assessment [15,35,37].

Analysis of genetic information has demonstrated that CKD is not uniform and is instead characterized by many different biological variations, as defined by the differences in genetic location and impact on baseline kidney function, albuminuria, and disease progression [1,6]. The identification of subtypes suggests that therapeutic effects will be maximized by aligning treatments with genetically defined subtypes of kidney disease, allowing clinicians to implement precision medicine approaches to treatment in nephrology. In this context, patients with genetic predisposition to accelerated declines in eGFR may benefit most from an approach designed to repair tubular injury and promote tubular health, while individuals with albuminuric CKD will likely benefit more from therapies directed at the glomeruli or endothelium.

Finally, the integration of GWAS with functional genomics, including kidney-specific eQTLs, chromatin interaction maps, and experimental validation, has begun to bridge the gap between statistical associations and actionable biology [13,15]. When combined with evidence that genetically supported targets are more likely to succeed clinically [35], this framework provides a rational and data-driven foundation for the discovery of CKD drugs. As these integrative pipelines mature, genetically informed target prioritization is expected to reduce attrition and accelerate the development of mechanism-based therapies that modify the natural history of CKD rather than treating late-stage consequences.

## 7. Challenges and Future Directions

Despite significant advances in GWAS, functional genomics, and experimental validation, the translation of genetic findings into a comprehensive, clinically relevant understanding of CKD is still limited. These limitations encompass barriers to obtaining accurate data and data diversity, understanding phenotypes and their relationship to the genetics of CKD, and the development of diseases. Above all, these obstacles need to be removed to take full advantage of precision nephrology. Additionally, several limitations interact with each other; thus, addressing an obstacle (e.g., accessibility and diversity of data) will deliver more significant benefits in other areas [1,15,37]. Therefore, collaborative, multidisciplinary efforts that are well-coordinated to remove limitations are essential.

A central limitation of CKD genetics is the mismatch between the scale of GWAS and the depth of kidney-specific functional data. While GWAS now routinely includes hundreds of thousands to over a million individuals, kidney-relevant molecular datasets, such as eQTLs, chromatin accessibility maps, and chromatin interaction data, remain comparatively small and are often derived from bulk tissues. This imbalance limits fine-mapping resolution, reduces the power for colocalization, and limits the detection of context-dependent regulatory effects, particularly those that are active only during injury or fibrosis [1,38]. The linkage of these approaches will require the systematic generation of kidney-specific multi-omic datasets at scale, including single-cell RNA-seq, single-nucleus ATAC-seq, spatial transcriptomics, and chromatin interaction maps, ideally generated from the same samples to enable integrative analyses [15,31]. Although statistical methods that incorporate functional priors can partially correct for the effects of limited sample sizes, they cannot fully substitute for increased biological resolution [15,38].

Another challenge is the lack of functional genomic data from diseased human kidneys. Most of the kidney maps available today are from healthy or only mildly affected tissues; however, CKD progression occurs through multiple dynamic processes, including inflammation, epithelial injury, and fibrotic remodeling. Although the introduction of single-cell and spatial studies has led to the acquisition of new knowledge surrounding disease-associated cell states, the size of patient samples is still small, and longitudinal sampling has not yet been regularly performed [15,31]. Expanding disease-state and longitudinal kidney datasets is critical for identifying genetic effects that occur only in pathological conditions. In particular, longitudinal sampling across disease stages and before and after therapeutic interventions would enable direct investigation of gene–environment and gene–treatment interactions [31,37,39,40]. Ethical and logistical restrictions associated with kidney biopsy highlight the need for innovative study designs, including the integration of nephrectomy specimens, transplant biopsies, and linked clinical–molecular cohorts [28,37].

Ancestry diversity is both a scientific and ethical priority. Most CKD GWAS and downstream functional resources remain heavily biased toward individuals of European ethnicity, despite the disproportionate burden of CKD in African, Hispanic/Latino, Indigenous, and South Asian populations. Limited ancestry diversity reduces discovery power, impairs fine-mapping, and undermines the generalizability of genetic findings and polygenic risk scores [41]. Encouragingly, recent multi-ethnicity GWAS have demonstrated that including diverse populations improves causal relationships by using differences in LD and can uncover ancestry-enriched risk variants relevant to CKD pathogenesis [1]. However, parallel efforts are needed to generate ethnicity-diverse kidney molecular datasets to avoid modifying the disparities downstream of GWAS. Without corresponding diversity in functional genomics resources, advances in genetic discovery risk widening the existing inequities in precision medicine implementation [41,42].

Another obstacle to GWAS is the presence of phenotypic heterogeneity in CKD; while CKD is clinically based on a reduced eGFR and/or albuminuria, the presence of these markers often combines multiple disease processes that occur through different molecular mechanisms. For example, a participant’s creatinine-based eGFR is influenced by non-renal factors, such as muscle mass, and may not provide a complete picture of the underlying renal dysfunction. Similarly, albuminuria exhibits substantial intra-individual variability; therefore, a participant’s diagnostic category of CKD, end-stage kidney disease, etc., may misrepresent the true underlying etiology of the pathology. The different types of GWAS signals produced by these multiple disease mechanisms make biological interpretation difficult. The recent use of longitudinal phenotypes (e.g., eGFR slope, progression to kidney failure) and investigation of CKD using etiology-specific datasets represents progress, but the studies continue to be limited by methodology [43] and limited power of the analyses, reflecting the more general problem of translating genetic variation into phenotypes across complex human diseases [38]. For continued progress, it is necessary to integrate genetic data with richer phenotypic information obtained from electronic health records, imaging, histopathology, and circulating biomarkers to develop biologically coherent subtypes of CKD [15,37,43].

To address many of the challenges identified in CKD research today, large-scale data sharing and the continued development of international consortia and disease-specific biobanks are required. The success of GWAS in CKD has been due to collaborations among researchers that allowed them to combine their data on a global scale. However, similar collaborative efforts do not yet exist for kidney-specific functional genomics, longitudinal phenotyping, or molecular profiling of the disease state. Therefore, integrating CKD biobanks with the ability to link genetic information to in-depth clinical phenotyping, longitudinal tracking, histopathology, imaging, and kidney-derived multi-omics will be a major step forward in providing robust causal inferences and reproducibility. These infrastructures should also provide consistent phenotype definitions to maximize the power of longitudinal and progression-related studies and allow for the systematic evaluation of gene–environment and gene–treatment interactions. Furthermore, to maximize the equitable representation of underrepresented populations and the broad distribution of precision nephrology’s benefits, these efforts must include the creation of equitable data-sharing policies and inclusive consortium governance. The development and application of polygenic risk scores (PRS) related to CKD suffer from additional conceptual challenges besides simply representing diverse ancestry. Ancestry diversity may be critical for mitigating biases and inequitable performance, but evidence suggests that the risk for CKD is also very dependent upon the context within which the genetic inheritance occurs. In particular, CKD risk may develop differently, change over the course of the disease, or reflect the genetic architecture linked to a person’s specific disease process at onset. Future PRS models may not be suitable when operating from globally created PRS models. Future models should consider the combination of ancestry, disease stage, the impact of comorbid disease states (such as diabetes), and the impact of environmental exposure on the CKD patients and PRS models, to be used to develop or stratify the phenotype training set, to provide a better clinical context for the results. Additionally, as trajectory information becomes available to PRS models based on disease progression or tracks phenotype progressions, PRS may become more clinically relevant in identifying patients at the highest risk for rapid CKD advancement or those with particular pathways of disease. However, to develop a comprehensive model of CKD risk and disease, large and harmonized datasets that provide longitudinal follow-up and validation for the clinical context and ethical use are required.

In conclusion, the clinical application of genetic insights faces additional obstacles related to discovery and interpretation. At present, polygenic risk scores for CKD explain a limited percentage of total CKD risk and offer limited clinical usefulness, except for research use [41,42]. This issue serves to illustrate the wider issues related to translating genetic variation into phenotype and clinical action [37]. Additionally, practical genetic results must be accompanied by effective therapeutic options to make any clinical use of them justifiable. Continued integration of GWAS with functional genomics, experimental validation, and drug discovery pipelines will, therefore, be essential to move from association to intervention [15,35]. As these efforts mature, future directions in CKD genetics will increasingly emphasize clinical stratification, therapeutic targeting, and equitable translation into patient care [1,35,37].

## 8. Conclusions

GWAS have transformed the understanding of CKD with an unbiased approach to identifying and understanding the genetics that contribute to kidney function, damage, and progression. Early GWAS showed that CKD is a polygenic disease. They also revealed new biological pathways that had not been emphasized in previous studies using candidate genes as starting points.

By integrating GWAS with other phenotypes across time (longitudinal data), across many different ethnicities (multi-ancestry GWAS), and complementary biomarker analyses, we now have a much clearer understanding of the genetics of kidney disease and the importance of separating the onset of CKD from its progression. The current research combines fine-mapping, eQTLs, single-cell transcriptomics, spatial transcriptomics, and experimental validation to define the GWAS loci with respect to the causal genes and mechanisms. This work also highlights the context-specific actions of the majority of the non-coding variants identified in GWAS. Specifically, these variants do not produce an effect unless or until there is injury, inflammation, or fibrosis. The results of these studies also underscore the importance of using molecular data related to disease and the limitations of bulk tissue analyses at steady state when studying kidney disease.

In addition to providing important biological insights into disease processes, human genetics creates an opportunity for the translational discovery of new therapies. It provides a rational framework for therapeutic prioritization in CKD, where trial failure remains high. Integrative genomics enables more selective and precise identification of therapeutic targets and assessment of safety by linking genetic relationships to causal genes, pathways, and cell types, allowing researchers to classify patients into biology-based models.

However, despite the significant progress made in this area, many significant obstacles remain. Together, these advances position human genetics as a central framework for mechanistic discovery, therapeutic prioritization, and precision nephrology. Therefore, overcoming these limitations will require a collaborative and coordinated approach to create various molecular datasets (e.g., cell-resolved and disease-state datasets), develop new methodologies, and perform further experimental validation. Furthermore, it is of critical importance that any progress made in genetics related to CKD is shared across all groups of individuals who are disproportionately affected by CKD.

In conclusion, human genetics research has transitioned from identifying regions of chromosomes associated with CKD to a comprehensive framework for mechanistic understanding, prioritization of therapeutics, and development of individualized approaches in nephrology for patients with CKD. The continued integration of large-scale genetic studies with functional genomics and experimental models will greatly facilitate the transition from genetic association to treatment intervention, allowing the development of disease-modifying therapies that target the underlying biological causes of CKD. Looking forward, continued integration of large-scale human genetics with kidney-specific functional genomics, longitudinal clinical data, and collaborative global research infrastructures holds the promise of transforming CKD research from descriptive association studies into a truly mechanistic and predictive discipline, ultimately enabling precision nephrology approaches that prevent disease progression and improve outcomes for patients worldwide.

## Figures and Tables

**Table 1:** Post-GWAS approaches for causal gene identification in chronic kidney disease.

Approach	Primary Objective	Key Data Sources	Strengths	Limitations	Key CKD Examples	Reference
Statistical fine-mapping	Identify likely causal variants within GWAS loci	GWAS summary statistics, LD reference panels	Reduces candidate variants; probabilistic framework	Limited by LD, sample size, ancestry bias	Fine-mapping of eGFR and eGFR decline loci	[1,2,25,26,27]
Functional fine-mapping	Prioritize regulatory variants using functional annotations	Chromatin accessibility, histone marks, TF binding	Incorporates biological context; improves prioritization	Dependent on tissue relevance and annotation quality	Enrichment of eGFR variants in tubular enhancers	[1,13,25]
Kidney eQTL analysis	Link variants to gene expression changes	Bulk kidney RNA-seq, compartment-specific eQTLs	Direct regulatory evidence; tissue specificity	Limited power; bulk tissue averaging	Identification of MANBA, DAB2	[15,16]
Compartment-resolved eQTLs	Resolve regulatory effects in the kidney compartment	Microdissected glomerular and tubulointerstitial tissue	Reveals compartment-specific regulation	Limited sample size	Tubulointerstitial enrichment of CKD loci	[15]
Colocalization (e.g., eCAVIAR)	Test shared causal variants between GWAS and QTLs	GWAS + eQTL summary statistics	Distinguishes true regulation from LD	Sensitive to LD structure and sample mismatch	PGAP3, KNG1, MANBA	[3,4,16]
TWAS	Associate genetically predicted expression with disease	GWAS + expression reference panels	Identifies distal candidate genes	Confounded by co-regulation; no formal causality	Kidney-function TWAS hits	[5,16]
Single-cell transcriptomics	Map genetic risk to specific cell types	scRNA-seq, snRNA-seq	High cellular resolution; disease-state mapping	Sparse expression; limited genetic integration	Proximal tubule enrichment for eGFR	[28,29,30,31]
Spatial transcriptomics	Map gene expression to anatomical niches	Spatial RNA profiling	Links genetics to pathology microenvironments	Lower gene coverage; cost	Fibrotic tubulointerstitial niches	[30,31]

Abbreviations: GWAS, genome-wide association study; LD, linkage disequilibrium; eQTL, expression quantitative trait locus; TWAS, transcriptome-wide association study; TF, transcription factor.

## Data Availability

No new data were created or analyzed in this study.

## Abbreviations

The following abbreviations are used in this manuscript:

ADPKD	Autosomal dominant polycystic kidney disease
AKI	Acute kidney injury
CKD	Chronic kidney disease
eGFR	Estimated glomerular filtration rate
eGFRcrea	Estimated glomerular filtration rate based on serum creatinine
UACR	Urinary albumin-to-creatinine ratio
GWAS	Genome-wide association study
LD	Linkage disequilibrium
QTL	Quantitative trait locus
eQTL	Expression quantitative trait locus
sQTL	Splicing quantitative trait locus
pQTL	Protein quantitative trait locus
TF	Transcription factor
TWAS	Transcriptome-wide association study
scRNA-seq	Single-cell RNA sequencing
snRNA-seq	Single-nucleus RNA sequencing
